# To Clone or Not To Clone: Method Analysis for Retrieving Consensus Sequences In Ancient DNA Samples

**DOI:** 10.1371/journal.pone.0021247

**Published:** 2011-06-27

**Authors:** Misa Winters, Jodi Lynn Barta, Cara Monroe, Brian M. Kemp

**Affiliations:** 1 School of Biological Sciences, Washington State University, Pullman, Washington, United States of America; 2 Department of Anthropology, Washington State University, Pullman, Washington, United States of America; 3 Department of Anthropology, University of California-Santa Barbara, Santa Barbara, California, United States of America; Natural History Museum of Denmark, Denmark

## Abstract

The challenges associated with the retrieval and authentication of ancient DNA (aDNA) evidence are principally due to post-mortem damage which makes ancient samples particularly prone to contamination from “modern” DNA sources. The necessity for authentication of results has led many aDNA researchers to adopt methods considered to be “gold standards” in the field, including cloning aDNA amplicons as opposed to directly sequencing them. However, no standardized protocol has emerged regarding the necessary number of clones to sequence, how a consensus sequence is most appropriately derived, or how results should be reported in the literature. In addition, there has been no systematic demonstration of the degree to which direct sequences are affected by damage or whether direct sequencing would provide disparate results from a consensus of clones.

To address this issue, a comparative study was designed to examine both cloned and direct sequences amplified from ∼3,500 year-old ancient northern fur seal DNA extracts. Majority rules and the Consensus Confidence Program were used to generate consensus sequences for each individual from the cloned sequences, which exhibited damage at 31 of 139 base pairs across all clones. In no instance did the consensus of clones differ from the direct sequence. This study demonstrates that, when appropriate, cloning need not be the default method, but instead, should be used as a measure of authentication on a case-by-case basis, especially when this practice adds time and cost to studies where it may be superfluous.

## Introduction

The ability to study DNA from organisms that have been long dead [i.e. ancient DNA (aDNA)], has led to numerous insights into the evolutionary history of humans, animals, plants, and even microorganisms [1], [2], [3], [4], [5], [6], [7], [8], [9], [10], [11], [12], [13], [14]. The strength of aDNA evidence is affected, however, by its challenging retrieval and authentication, principally as a result of postmortem damage. Degradation by nucleases, oxidation, deamination, depurination, and background radiation lead to destabilization and breaks in DNA strands [15] leaving aDNA template molecules typically short in length with chemically modified (i.e. “damaged”) nucleotide positions [16], [17]. Consequently, aDNA studies are prone to contamination from “modern” DNA sources that can completely out-compete endogenous DNA in polymerase chain reaction (PCR) amplification [18]. These problems are not unique to the aDNA field, but are also encountered in forensic research where degraded remains and sample mixtures are common [19], [20].

Troubled by the overwhelming lack of standards followed by aDNA practitioners that presented at the 5^th^ International Ancient DNA Conference in 2000, Cooper and Poinar [21], published a very timely opinion piece in *Science* that outlined a list of criteria that should be followed in order to authenticate aDNA evidence for publication [21]. The recommendations of Cooper and Poinar [21] have had a profound impact on the field both positive and, and in some cases, negative. For example, reviewers have rejected manuscripts written by authors that did not follow each and every recommendation of Cooper and Poinar [21], referring to them as “classical stringent standards” [22], despite the fact that subsequent research clearly showed that the recommendations of Cooper and Poinar [21] alone can not authenticate aDNA evidence [22], [23]. Additionally, some of their criteria such as amino acid racemization (AAR) have been discounted as a predictor of DNA preservation [24], [25], while in contrast, critical decontamination methodologies [e.g. 18] were never “required”. Unfortunately, one of the most critically important points made by Cooper and Poinar [21], that data produced need to make sense, rarely generates much attention.

This study focuses on the fifth recommendation of Cooper and Poinar [21], which states “Direct PCR sequences must be verified by cloning amplified products to determine the ratio of endogenous to exogenous sequences, damage-induced errors, and to detect the presence of numts. Overlapping fragments are desirable to confirm that sequence variation is authentic and not the product of errors introduced when PCR amplification starts from a small number of damaged templates”. Since publication of Cooper and Poinar's [21] critique, cloning has become a common practice, yet no standardization has emerged regarding the number of clones required to produce an appropriate consensus, or how to evaluate the validity of the clones that are generated. In addition, there has been no systematic demonstration of the degree to which direct sequences are affected by damage or whether direct sequencing would provide disparate results from a consensus of clones. To address these issues, aDNA was extracted from the remains of five ∼3,500 year old northern fur seals (*Callorhinus ursinus*). Results from direct sequencing and cloning of a portion of the mitochondrial cytochrome B gene were compared following a simple majority rules approach. Furthermore, we evaluated the usefulness of the Consensus Confidence Program (CCP) [26] in deriving consensus sequences.

### Background: Variability and Inconsistency in the Cloning of aDNA

To illustrate the variability of cloning methodologies, Table 1 summarizes the cloning practices of twenty-nine aDNA studies published in various journals over a sixteen-year period (1994–2010). The data indicate tremendous inter-study variability, with researchers reporting as few as two clones to over 100 per amplification. Some researchers chose to clone only a subset of samples from a given archaeological site to evaluate sequencing fidelity [e.g. 27], which suggests that they believe that taphonomic processes are uniform across a site. This is in stark contrast to the notion that sample specific qualities, such as the copy number of target DNA, should dictate the need to practice cloning, namely the preserved copy number of target DNA [2], [28].

**Table 1:** Example of studies that utilized cloning in the study of aDNA sorted by year of publication.

		Number of Clones Sequenced											
Study	Comparison to Direct Sequence?	2	3	4–5	6–8	9–11	12–20	21–40	41–60	61–80	81–100	101+	Species/Samples
Handt et al [40]	N						X						Human (Tyrolean Iceman)
Handt et al [28]	N		X	X	X								Human
Krings et al [4]	N			X	X	X	X						Neanderthal
Poinar et al [35]	N				X	X	X						Ground Sloth
Krings et al [44]	N				X	X	X						Neanderthal
Ovchinnikov et al [45]	Y		X	X									Neanderthal
Hofreiter et al [15]	N				X	X							Cave Bear
Loreille et al [46]	Y		X	X	X								Cave Bear and Brown Bear
Hofreiter et al [47]	N		X	X	X	X	X						Cave bear
Monsalve et al [48]	Y			X	X								Human
Caramelli et al [49]	N			X	X	X							Human
Orlando et al [50]	N	X	X	X	X								Woolly rhinoceros
Poinar et al [51]	N				X	X	X						Sloth
Gilbert et al [52]	N			X	X	X	X						Human
Bouwman and Brown [53]	N			X									Humans, Syphilis
Haak et al [54]	N			X	X	X	X						Human
Jae-Hwan et al [55]	N		X										Cows
Karanth et al [56]	N		X	X									Lemurs
Malmstrom et al [57]	N								X				Human, Dog
Salamon et al [37]	N			X	X								Cat, Penguin, Human
Binladen et al [58]	Y			X		X							Woolly Rhinoceros, Lion, Pig, Moa
Gilbert et al [59]	N				X	X			X	X			Human
Orlando et al [60]	N				X		X						Neanderthal
Krause et al [33]	N		X		X		X				X	X	Neanderthal
Kuch et al [32] *	N			X	X								Human
Green et al [61]	N											X	Neanderthal
Helgason et al [62]	N	X	X	X	X	X	X	X	X	X			Human
Kuhn et al [27]	Y					X							Caribou
Lari et al [63]	N						X	X					Neanderthal

Categories for number of clone sequences were arbitrarily chosen.

*estimated number of clones from Figure 3 [32].

Also troubling, are studies that report the number of clones sequenced yet do not publish the results [see for example 29], [30], [31] or provide readable sequence data [32]. As a result, it is impossible to evaluate the strength of the data generated, despite the fact that these studies followed the cloning recommendation. This suggests that some reviewers are not evaluating the cloning data itself, but are satisfied merely with the fact that the technique was used during the experimental process. It also means that authors need to be more responsible in clearly reporting their data.

Another major problem with current cloning practices relates to how consensus sequences emerge from the cloned sequence data. Methods for determining consensus sequences are highly variable and lacking standardization. Most studies listed in Table 1 took a majority rules approach to building consensus sequences. This supposition suggests that minority sequences, based solely on their minority status within a pool of clones, represent contaminating and/or chemically modified (i.e. damaged) template molecules. Alternately, in an investigation studying DNA extracted from hominid specimens from Southern Siberia, Krause and colleagues [33] used the minority status (2 of 104 clones) of Neanderthal-like mitochondrial DNA (mtDNA) sequences to initially support identification as non-human. While Krause and colleagues [33] rightly used additional means to authenticate their species identification, this serves as a reminder that the use and interpretation of cloning, and its results, is variable and that the most important criterion is that the data make sense.

Dealing with highly damaged DNA also raises the question of whether a cloning consensus can and should be combined from two separate extracts. When reactions start from a separate pool of template molecules extracted on different occasions, it is preferable to generate a consensus from the extracts separately and use each as independent confirmation of the other. When low copy number and damage render this strategy impossible, another extract attempt should be made to confirm the piecemeal consensus sequence. Reporting the ambiguities is an option if, after several attempts at confirmation, a consensus cannot be generated [28]. Ultimately, the act of cloning itself does not make the data generated any more authentic and the necessity of the technique and the validity of consensus sequences should be closely monitored on a case-by-case basis.

Despite methodological inconsistencies in the field, a glimmer of clarity was provided by Bower and colleagues [26]. These researchers created a freeware program called the “Consensus Confidence Program” which produces a consensus by calculating the percent probability that statistically each nucleotide occurs most frequently, at an individual position, with a confidence level between 70% and 95%. The program requires a minimum input of 12 clones to generate a consensus sequence. While this program is a tool that offers the means to standardize and produce statistically significant consensus results, it is important to highlight that it cannot “verify whether the consensus sequence is authentic” (pg. 2550).

Regardless of very strong encouragement for the use of cloning by aDNA researchers, there has been no systematic demonstration that directly sequenced PCR products would represent anything but the majority rules consensus of a number of clones [22]. The cloning recommendation of Cooper and Poinar [21] was adopted as a mandatory default technique by those in the aDNA field without critical evaluation. From the studies described in Table 1, one finds that in only five of the 29 studies did the researchers even compare cloned sequences to direct sequences. In none of these studies did the majority rules consensus sequence differ from the direct sequence.

An original goal of this study was to use published data to compare direct sequences with the consensus of a minimum of 12 clones as determined by the CCP [26]. This goal was unachievable as none of the five studies sequenced more than ten clones. Nevertheless, the data in the reviewed literature (Table 1) suggest that cloning aDNA amplicons is not necessary in all cases, especially when this practice adds time and cost to studies where it may be superfluous.

Recommendations for maintaining authenticity in aDNA studies are always appreciated, but it may not necessarily be true that cloning is the only way to generate accurate sequence results. Rather, directly sequencing amplicons from independent amplifications and extractions may be sufficient. The goal of this study is to begin the systematic determination of whether a difference, if any, exists between cloning and direct sequencing in order to generate an aDNA consensus sequence. Working specifically with non-human, non-domesticate animal samples decreases the probability that contamination has contributed to these results [18], [34].

## Results

**Figure 1:**
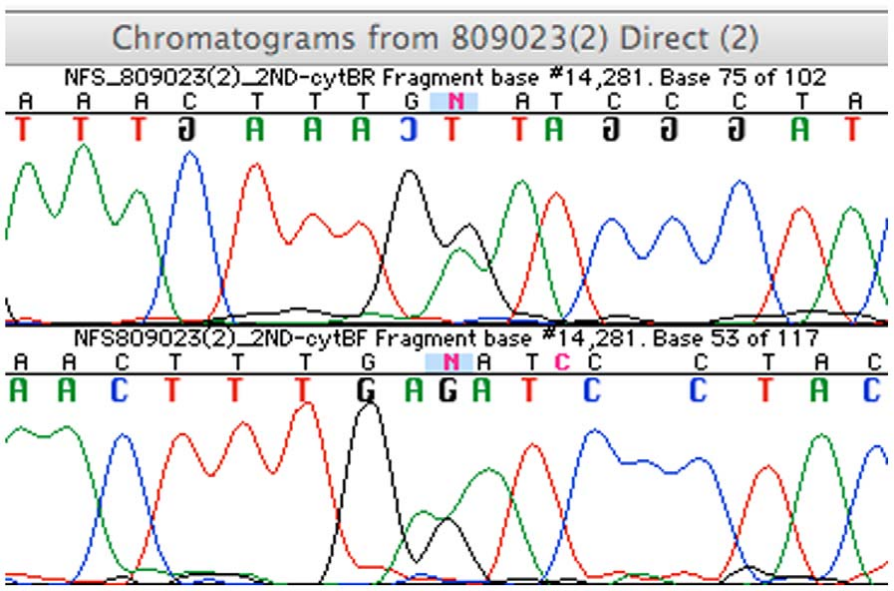
Chromatogram for site 14281 on sample 809023.

Deviations among the clones from the consensus were observed at a total of 31 sites within 150 cloned fragments across the five samples (Table 2, Figure S1). Single base polymorphisms that appear as “transitions” in the clones, when compared to the direct sequence, were recorded as damage. The majority of the damaged sites were C>T, which is indicative of deamination [15]. Sites with double peaked base pairs in the cloned sequences (designated as N) must represent errors that arose during colony growth or subsequent PCR (see Figure 1 for an example).

**Table 2:** Results from the sequencing and quantification of samples.

Sample	Copy #/µL	S.D.	Amplication #	Direct Sequence	Majority Rules	CCP	Clones
			**1**	14285 A	14285 A	14285 A	13:14285A; **1:14285N**
**809005**	**644**	**9.784**	**2**	14285 A	14285 A	14285 A	10:14285A; 1:14285A, **14293T**; 1:14285A, **14322A**
			**3**	14285 A	NA	NA	Not cloned
			**1**	Reference	Reference	Reference	14:Reference; **1:14285N, 14289N**; **1:14300N**
**809007**	**1737**	**332.96**	**2**	Reference	Reference	Reference	14:Reference; **1:14210A, 14227A, 14231A, 14247A, 14316T**; **1:14241T, 14244T, 14245T, 14246T, 14284T**
			**3**	Reference	NA	NA	Not cloned
			**1**	Reference	Reference	Reference	15:Reference; **1:14223G**
**809016**	**91**	**29.132**	**2**	Reference	Reference	Reference	15:Reference; **1:14334A**
			**3**	Reference	NA	NA	Not cloned
			**1**	Reference	Reference	Reference	10:Reference; **1:14210A, 14281A,14339N**; **1:14227A, 14265A, 14280A, 14304A**: **1:14281N**
**809023**	**35**	**3.868**	**2**	**14227N, 14231N, 14265N, 14269N, 14281N**	None	None: Did not meet 95% confidence	**5:14227A, 14231A, 14265A, 14269A, 14281A, 14304A, 14334A; 3:14227A, 14231A; 2:14210A, 14211A, 14265A, 14281A, 14299A, 14232A,14233A; 1:14227A, 14231A,14265A, 14269A, 14281A, 14304A; 1:14210A, 14211A, 14334A; 1:14265A, 14269A,14334A; 1:14280A, 14281A, 14299A, 14304A, 14334A; 1:14265A, 14269A, 14281A, 14304A, 14334A**
			**3**	Reference	NA	NA	Not cloned
			**1**	14285A	14285A	14285A	11: 14285A; **2:14227A**, 14285A; **1:14250N**, 14285A; **1:142545T**, 14285A; 1:14285A, **14334A**
**809032**	**115**	**42.496**	**2**	14285A	14285A	14285A	14:14285A; **1:14227A**, 14285A; **1:Reference**
			**3**	14285 A	NA	NA	Not cloned

Samples were sequenced from nps 14207–14345, relative to a complete mtDNA genome, NC_008415 [42], and polymorphisms listed (not in bold) are relative to that reference sequence. Consensus sequences have been submitted to Genbank and assigned accession numbers HQ95713-HQ95717. For the clone category, results should be read as follows: the first number refers to the number of clones that have the damage/error, the second number provides the position in the sequence followed by the letter of the base pair that is now seen (e.g. 13:14285A reads as 13 clones with an adenine present at site 14285). Quantification results represent the average copy number over duplicate qPCR reactions and the standard deviation is reported. Note that qPCR results are indicative of the relative DNA level, but should not be taken as exact quantification.

None of the five samples showed any difference between the direct sequence, the majority rules consensus, and the consensus as determined by the CCP (Table 2). The sole exception to this finding is the second transformation of sample 809023, where a majority rules consensus could not be determined because the most common haplotype was present in only 5 of 12 clones (41.7%). Similiarly, a 95% confidence consensus from the CCP could not be determined due to the high number of unique sequences among the clones. The direct sequence for this transformation does however, accurately reflect the mix of cloned sequences. That is, the competition of peak intensities at the N sites in the direct sequence correlate with positions in the clones that reveal a substantial mix of adenines and guanines. For example, at site 14281, 10 out of 15 clones show an A instead of a G (Table 2, Figure S1) and the chromatogram shows competing A and G peaks (see Figure 1). In this case, the third independent PCR amplification was consistent with the first PCR amplification and first transformation (Table 2, Figure S1).

The quantification of samples shows a diverse range of average copies of mtDNA per microliter from 35 (SD 4) to 1737 (SD 333) (Table 2).

## Discussion

While we have chosen here to work with non-human, non domestic animal samples, this is the first study to demonstrate that directly sequencing aDNA can provide the same data as taking a consensus of clones (as assessed by a majority rules approach, the CCP, or both), but it is prudent to mention that our results cannot be extrapolated across all studies. For instance there are cases in aDNA research where cloning is an absolute necessity. Without relying on the capacity of next generation sequencing, cloning would be, for example, the only means of reconstructing ancient diets from DNA preserved in coprolites [11], [35], or studying a mixture of DNA extracted from soil [9], [10] or ice samples [8]. The reason that cloning is essential in these cases is that their goal is to observe as many unique molecules as permitted, not to reach a consensus sequence from a pool of clones. In contrast, the focus of this initial study was deriving a consensus sequence from endogenous molecules from single individuals.

Cloning would also be necessary if the goal of a study is to derive an aDNA sequence from a heavily contaminated sample that cannot be decontaminated prior to DNA extraction. For example, there has been no demonstration that human coprolites can be efficiently decontaminated, which is why cloning was necessary to conclude that the coprolites excavated from Paisley Caves were produced by the occupants of the caves [36]. However, this conclusion was not drawn from taking a consensus of a pool of clones, rather it relied on knowledge about the mtDNA mutations exhibited by the first Americans, relative to those exhibited by non-Native Americans. In contrast, if a sample can be sufficiently decontaminated [e.g. bone or tooth samples [18], [37], [but see 38]] cloning may be less necessary in deriving an individual sequence. While the experiment was not conducted, it would have been very interesting if Krause and colleagues [33] had decontaminated a piece of Neanderthal bone, and extracted and analyzed this in parallel with the samples that they did not decontaminate. Then the results from the decontaminated bone could be compared against the 98% contamination they observed in the clones (102 of 104) of their experiment. Again, the experiments in this study were not conducted to address this issue; therefore, the results are not directly applicable to either of these scenarios. Future studies that explore the relationship between decontamination and cloning are necessary.

Cloning remains an appropriate and reliable method for obtaining aDNA sequences, given that this practice has the potential for showing the composition of a mixed PCR reaction (whether the heterogeneity of molecules arose from damage or contamination). However, as shown here in the second amplification of sample 809023, direct sequencing also permits one to see that the authenticity of a sequence is compromised by having started from a highly heterogeneous pool of molecules (i.e. when double peaks are present in the chromatograms). This amplification of the sample shows that even with competing damage, both approaches will yield the same result, and would require an additional amplification to reach a consensus for the sequence. Given the results presented, we argue that cloning should not serve as the default first step method for obtaining consensus sequences from aDNA samples, as has become commonplace in the field. This is especially true considering that direct sequencing is more time and cost efficient and, thus, could hasten discovery and publication.

While there is a general “rule” in the aDNA field that one should be suspicious of sequences initiated from a pool of less than 1000 template molecules [2], our study has shown that even very low copy number samples [35 copies/µL (SD 4)] can provide reliable direct sequences. This “1000 molecule rule” originated from a study conducted by Handt and colleagues [28], who actually stated “A minimum of 100–1,000 molecules per amplification” (pg. 375) may be needed to get around the problems of sporadic contamination and/or damaged template molecules. As this cut off was determined with much cruder methods than are available today, we suggest that the relationship between the number of starting template molecules in a PCR and the reliability of the resulting sequence (whether produced directly or from a consensus of clones) needs re-evaluation. We anticipate that the repeatability of data will be more crucial to determining authenticity than starting template molecule copy numbers [39], an expectation which is supported by the reliability of the sequence derived from our lowest copy number sample (809023).

Ancient DNA research is positioned to continue to provide answers to questions of the past but, as most practitioners in the field recognize, collection and authentication of results will always be a challenge. With all the problems and circumstances associated with aDNA, researchers must be proactive in minimizing inaccurate results that can lead to dubious claims. While the recommendations of Cooper and Poinar [21], or any other list of recommendations, were created as well intentioned advice for ensuring accurate results, they should not act as a simple checklist for researchers to follow and reviewers to note [23]. We do not outright reject these recommendations because in practice they are aimed at reducing contamination and strengthening evidence that the molecules are, in fact, ancient. However, following the rationale outlined by Gilbert and colleagues [23] and Kemp and Smith [22], and supported by the data presented here, we disagree that protocols in the aDNA field should be dictated by a methods checklist. We recommend that researchers be as explicit as possible in describing their methods as well as their rationale for using them. It is appropriate for researchers to state their reason for cloning, other than just to satisfy the requirements suggested by Cooper and Poinar [21] [for example was done by 28], [40]. This allows the reader to better understand the characteristics of the sample and the critical analysis that contributed to making methodological choices. For example, if the research question relies on the knowledge that a PCR reaction began from a heterogenous pool of molecules, cloning would be an appropriate method to confirm this. However, as demonstrated here, generating a sequence from an ancient sample does not require cloning and, as such, the method need not serve as the default approach.

Ancient DNA data should be evaluated according to the specific methods used to generate them, paying particular attention to the degree to which the data make sense. A cognitive approach to aDNA is necessary for assessing the reliability of results. Each study has specific problems and criteria that need to be considered in order to advocate reliable data. The “Key questions to ask about ancient DNA” (pg 543) as suggested by Gilbert and colleagues [23] throws out the idea of a requirements checklist and instead proposes that readers, reviewers, and authors alike analyze whether or not the results make sense within the context of the study. Similarly, the results presented here underscore the point that rather than employing a methods checklist, reviewers need to more critically appraise the data that are presented in a study in order to judge the quality of research.

## Materials and Methods

Between 0.40 and 0.77 g of bone was removed from the distal end of five northern fur seal (*Callorhinus ursinus*) rib bones (samples are designated 809005, 809007, 809016, 809023 and 809032) using a new dremmel blade for each sample. These samples were excavated from the Amaknak Bridge Site in Unalaska, AK and date to approximately 3,500 years before present (YBP) [41]. All DNA extractions and PCR set-up were conducted in the Kemp Ancient DNA Lab at Washington State University. The samples were submerged in 6% w/v sodium hypochlorite for 15 min and rinsed twice with DNA free H_2_O to remove surface contamination [18]. DNA was extracted following Kemp and colleagues [13] except that the original volume of sample 809023 in EDTA was split in half before the phenol/chloroform step. A 181 base pair (bp) portion of the cytochrome B gene spanning nucleotide positions (nps) 14185–14365 [relative to a complete mtDNA genome, NC_008415 [42]] was PCR amplified with primers: CytB-F CCAACATTCGAAAAGTTCATCC and CytB-R GCTGTGGTGGTGTCTGAGGT [43] for quantification by Real Time PCR and for use in direct sequencing and cloning.

Quantification PCRs were performed on sample extracts in an Applied Biosystems 7300 Real Time PCR System using a MAR-labeled probe: 5′-CATTAACAGCTCGCTC-3′ (Allelogic). Each 25 µL reaction contained 0.24 mM dNTPs, 1× PCR Buffer, 1.5 mM MgCl_2_, 0.4 µM of each primer, 0.24 µM probe, 0.5 µM ROX reference dye, 0.75 U of Platinum *Taq* polymerase (Invitrogen™), and 5.0 µL of extract at full concentration, 20%, and 10% to determine levels of inhibition and ensure accuracy of copy numbers. Cycling was performed with an initial 10 minute hold at 95°C followed by 50 cycles of 15 seconds at 95°C and 60 seconds at 55°C. A minimum of 4 negative template controls were included on each 96-well plate to monitor contamination in reagents and ROX-labeled passive reference dye was included to correct for variation in well-to-well background fluorescence. Amplification curves were analyzed with the automatic baseline feature of the 7300 System SDS software (Applied Biosystems) with an empirically determined threshold of 0.05. Calibration curves were generated from a freshly prepared serial dilution series of standard DNA amplified from modern northern fur seal whole genomic DNA extract. Slopes of the calibration curves were used to calculate assay efficiencies (%PCR efficiency = (10^(−1/slope)^−1)×100) and all were required to meet an efficiency >87% with R^2^>0.996 for data inclusion. Analyzed data were exported from the 7300 SDS software into a CSV file (comma delimited) for secondary analysis and formatting in Microsoft® Excel 2007.

Amplifications for direct sequencing and cloning contained 0.32 mM dNTPs, 1× PCR Buffer, 1.5 mM MgCl_2_, 0.24 µM of each primer, 0.3 U of Platinum *Taq* polymerase (Invitrogen™), and 3.0 µL of DNA template in 30 µL reactions. These reactions were subjected to 60 cycles of PCR as follows: 3 min denaturing at 94°C, followed by 15 second holds at 94°C, 55°C, and 72°C, with a final 3 min extension period at 72°C. Negative control amplifications were carried out to detect potential contamination. Two independent PCR amplifications from each of the five extracts were submitted for direct sequencing. One microliter from each amplification was then cloned using a TOPO® TA cloning kit and TOP10 competent cells (Invitrogen™) following manufacturer's instructions with the exception that reactions were scaled to one quarter. A minimum of 16 white colonies were selected from each sample transformation and underwent colony PCR using the CytB primers for the first transformation and M13 primers for the second transformation. Colony PCRs were the same as above except they were prepared for a 15 µL reaction with 1.5 µL of DNA template, and the M13 primers were cycled with an annealing temperature of 58°C. Control plates and transformation of PCR negatives were used to ensure cell competency and PCR amplifications free of contamination. Clones containing the transformed vector were then sequenced at a minimum of 13 clones per sample. All amplicons were prepared for sequencing and purified using a Multiscreen PCR_μ96_ filter plate (Millipore). Amplicons were brought to a volume of 100 µL, using dH_2_0, before transfer to the filter plate. After vacuuming, 25 µL of dH_2_0 was added to each well, followed by 30 minutes of shaking at 350 rpms. Direct sequencing was performed in both directions at the DNA Analysis Facility at Yale University. Sequences were aligned to a complete northern fur seal mtDNA reference sequence [Genbank accession number NC_008415 from 42] using Sequencher® 4.8.

As mtDNA does not undergo recombination, the majority rules consensus sequence was determined to be the haplotype present in greater than 50% of the clones. Cloned sequences from the five samples were analyzed by the CCP [26] to determine percent confidence and any variation(s) from the majority rule consensus sequence. The direct sequence was then compared to each consensus sequence.

As an additional control, a third PCR amplification was directly sequenced as described above, but not cloned, for comparison to the first two direct sequences and to that of the consensus sequences determined from the sequenced clones from these PCRs.

## Supporting Information

Figure S1 **Cloned sequencing results.**
(XLS)
